# Computational Tools to Assist in Analyzing Effects of the *SERPINA1* Gene Variation on Alpha-1 Antitrypsin (AAT)

**DOI:** 10.3390/genes15030340

**Published:** 2024-03-06

**Authors:** Jakub Mróz, Magdalena Pelc, Karolina Mitusińska, Joanna Chorostowska-Wynimko, Aleksandra Jezela-Stanek

**Affiliations:** 1 Tunneling Group, Biotechnology Center, Silesian University of Technology, Krzywoustego St. 8, 44-100 Gliwice, Poland; k.mitusinska@tunnelinggroup.pl; 2 Department of Genetics and Clinical Immunology, National Institute of Tuberculosis and Lung Diseases, 26 Plocka St., 01-138 Warsaw, Poland; m.pelc@igichp.edu.pl (M.P.); j.chorostowska@igichp.edu.pl (J.C.-W.)

**Keywords:** *SERPINA1*, alpha-1 antitrypsin, in silico analysis, computational tools, bioinformatics, next-generation sequencing

## Abstract

In the rapidly advancing field of bioinformatics, the development and application of computational tools to predict the effects of single nucleotide variants (SNVs) are shedding light on the molecular mechanisms underlying disorders. Also, they hold promise for guiding therapeutic interventions and personalized medicine strategies in the future. A comprehensive understanding of the impact of SNVs in the *SERPINA1* gene on alpha-1 antitrypsin (AAT) protein structure and function requires integrating bioinformatic approaches. Here, we provide a guide for clinicians to navigate through the field of computational analyses which can be applied to describe a novel genetic variant. Predicting the clinical significance of *SERPINA1* variation allows clinicians to tailor treatment options for individuals with alpha-1 antitrypsin deficiency (AATD) and related conditions, ultimately improving the patient’s outcome and quality of life. This paper explores the various bioinformatic methodologies and cutting-edge approaches dedicated to the assessment of molecular variants of genes and their product proteins using *SERPINA1* and AAT as an example.

## 1. Introduction

The development and application of accurate and reliable computational tools to assess the outcome of single nucleotide variants (SNVs) is of great importance not only in the field of bioinformatics, but also for clinicians. Understanding the significance of SNVs plays a crucial role in deciphering the molecular basis of diseases, population genetics, and personalized medicine. There is a great promise in steering therapeutic interventions and shaping the trajectory of personalized medicine in the foreseeable future, based on the molecular pathomechanism underlying genetic disorders [1]. A single nucleotide change in the DNA sequence may result in an alteration of the amino acid sequence (missense) or shortening of the product protein sequence (truncating, e.g., nonsense mutation), which can cause a spectrum of disorders, or may not affect the protein sequence at all. Moreover, the location of such point mutation is also crucial as it may impair the structure and function of the resulting protein. Other possible kinds of gene mutations may be more spectacular, covering small deletions or insertions of fragments of the gene sequence (indels), or even modifications of the number of gene copies (copy number variations, CNVs).

A synergy between bioinformatic researchers, molecular biologists, and clinicians is beneficial to accurately delineate the clinical significance of genetic variants in the *SERPINA1* (serpin family A member 1) gene. This, in turn, enables a refined and individualized approach to treatment strategies, especially pertinent in cases of alpha-1 antitrypsin deficiency (AATD) and related conditions. Pursuing such precision in medical interventions contributes significantly to the improvement of patient outcomes and an overall enhancement in the quality of life for those affected (see Section 4 “Clinical Consequences of Pathogenic Variants in *SERPINA1*”). In clinical practice, genetic diagnostics of rare monogenic disorders, such as AATD, is concentrated mainly on the study of the functional and clinical impact of SNVs, while the role of other types of variants remains less understood. A comprehensive grasp of the impact of SNVs within *SERPINA1* on the structure and function of alpha-1 antitrypsin (AAT) protein demands an integration of diverse bioinformatic methodologies.

This paper explores the various bioinformatics methodologies and cutting-edge approaches dedicated to assessment of the molecular variants of genes and their products—proteins. We review currently available computational tools which can be used to assess and report novel gene variants, focusing on their application and utility for the analysis of *SERPINA1* and its product AAT protein, which are associated with the etiology of AATD. AAT protein has an interesting and complex function mechanism, and its defects are related to various molecular pathomechanisms causing different clinical consequences, which makes AAT a valuable target for computational studies. Apart from the most common pathogenic changes (resulting in PI*Z or PI*S AAT protein variants), there is a steady increase in the number of rare and new molecular variants (mostly SNVs) of unknown significance reported in *SERPINA1* in relation to AATD phenotype, which demand thorough characterization and clinical interpretation. A brief description of the common and known rare variants of *SERPINA1* was provided elsewhere [2,3,4]. Our aim is to equip clinicians with a guide on the possibilities provided by computational tools and thus collaborations with computational scientists.

## 2. Molecular Variation: SNVs, CNVs, and Other

Molecular variation is fundamental for genetic diversity and associated with various genetic diseases, traits, and susceptibility to certain conditions. A molecular variant refers to a permanent change in DNA or RNA, involving an alternation of the coding or non-coding regions of the genome, quantity, or structure of the genetic material, which may or may not influence gene expression, protein structure, and function. Molecular variants have significant implications in human genetics, evolutionary biology, and medicine, as they can underlie genetic diseases, influence traits, disease susceptibility, drug responses, and evolutionary processes. Their role in human diseases is comprehensively studied in the context of medical genetics, and to develop diagnostic tools or targeted therapies.

Formerly, molecular variants were referred to as mutations; however, this term was not accurate, as variants may be disease-causing or benign, represent risk factors for certain conditions or constitute genetic biomarkers used to predict clinical benefits from targeted therapies, for example in cancer genetics. Molecular variants may be caused by spontaneous errors occurring on the genetic and epigenetic level during cellular processes or arise as an aftermath of the exposure to damaging agents in the environment. When germline (occurring in the germ cells), variants are hereditary and present in every cell in the body. Genetic disorders are mainly caused by germline variants, which can arise de novo (not present in the parents; except for ultra rare cases of germline mosaicism) or be passed down from an affected carrier (from parent to offspring). Somatic variants are generally not hereditary and are limited only to specific cells or organs (as they occur postzygotically or are acquired during a person’s life). Specific somatic changes may lead to carcinogenesis and underlie some genetic disorders. Postzygotic mutational events may also lead to the formation of genetically distinct cells (with different genotypes) within an organism, a phenomenon called mosaicism. It varies in terms of tissue distribution, where germline (or gonadal) mosaic variants occur in the germ cells (therefore, can be inherited), and somatic mosaic variants in other tissues. Also, a rare gonosomal mosaicism (combination of both types, germline and somatic) has been reported. Mosaicism may influence the phenotypic spectrum presented by an affected patient, for example by limiting disease symptoms to a specific organ or body part, and effect in a variable clinical expression of a disease [5,6].

Regarding population frequency of the molecular variants, only rare ones (with minor allele frequency, MAF, <1% in genetic databases, such as gnomAD) [7] are considered disease-causing. Variants common in the general population (MAF > 1%) are mainly benign genetic variation; however, some may influence the risk of developing certain disorders.

Among molecular variation we distinguish: (i) single nucleotide variants (SNVs), which include single nucleotide substitutions (effecting in missense/nonsense amino acid changes or splice-site alterations), and several to dozen nucleotide deletions, insertions and insertion-deletions (indels; mainly resulting in a frameshift) within a DNA sequence; (ii) copy number variation (CNV), comprising qualitative, structural variants in the genome, where segments of DNA are duplicated or deleted, resulting in an alteration in the number of copies of a particular genomic region. CNVs can vary in size, ranging from kilobases to megabases, and can involve genes, regulatory elements, or intergenic regions; and (iii) structural variation (SV) encompassing a broad category of genetic alterations involving large-scale changes in the structure of the genome, beyond SNV or CNV, such as deletions, duplications, inversions, translocations, and complex rearrangements of DNA segments, ranging in size from a few nucleotides to megabases, which can impact gene structure, function, and regulation [5,8,9].

Advanced technologies, such as Next-Generation Sequencing (NGS), have greatly facilitated the detection and characterization of molecular variants in the human genome. NGS data analysis from targeted gene panels enables mainly SNVs identification; exome- or genome-based studies enable the detection of both SNVs and CNVs changes, while SVs are evaluated only at the genome scale [10].

## 3. Molecular Variability of *SERPINA1*

AAT stands as a key antiprotease in the bloodstream and is a part of the serine protease inhibitor (SERPIN) superfamily [11]. Its encoding gene, *SERPINA1*, localized on chromosome 14q32.1, is notable for its extensive molecular variability [2,3]. The ClinVar database has documented around 400 molecular variants in *SERPINA1*, approximately 100 of which are deemed pathogenic or likely so according to globally recognized criteria, and over 150 variants are of uncertain or debated significance, necessitating further examination [12]. Additionally, the Human Gene Mutation Database (HGMD) has identified more than 140 known *SERPINA1* molecular variants associated with AATD and related disorders, significantly increasing the risk for liver and lung diseases [13]. SNVs are the most commonly identified alterations in *SERPINA1*, comprising mainly the missense changes and in some cases small deletions/duplications, while CNVs are rare. The most frequently encountered pathogenic AAT variant is the PI*Z, described as NM_000295.5:c.1096G>A p.(Glu366Lys) (rs28929474:C>T), according to the Human Genome Variation Society (HGVS) guidelines. It results in well-established pathogenicity, by inducing misfolding and subsequent polymerization of AAT in hepatocytes and other AAT-synthesizing cells [14,15]. However, with the increasing efficiency of diagnostic techniques, especially high-throughput sequencing, a collection of rare and novel variants with unknown clinical significance has been identified [16,17,18,19]. The unknown pathomechanism and the lack of clinical information on these variants greatly complicate genetic counseling and underscore the need for tools to assess their biological impact [16,20,21,22].

AAT is a glycoprotein consisting of 418 amino acids with a 24 amino acid signal peptide. Its structure comprises nine α-helices, three β-sheets, and multiple loops. One of the longest loops, which is also exposed to the solvent, is the reactive center loop (RCL) that modulates inhibitory activity of the protein. Methionine residue at position 358 (numbering according to the crystal structure of the native protein—PDB ID 3ne4; and 382 according to UniProt ID P01009), located within the RCL, determines protein’s specificity of interaction with the catalytic triad of serine proteases, mainly with human neutrophil elastase. The mature protein has molecular weight of about 52 kDa, 15% of which consists of carbohydrates. Carbohydrate chains are connected to the protein by three asparagine residues located at positions 46, 83, and 247 [23]. The mature protein is a single polypeptide chain; however, some of the molecular variants are known for their ability to polymerize. The most prevalent PI*Z variant of AAT is characterized by a single point mutation leading to an amino acid change of glutamate to lysine at position 342 (366 according to UniProt). This change causes a gap in a β-sheet of one AAT molecule, allowing cleaved RCL of the other molecule to insert itself into this gap, causing polymerization. This process, called loop-sheet polymerization, leads to accumulation of AAT in the hepatocytes [24].

Identifying all molecular variants in the *SERPINA1* sequence, including coding and non-coding areas, is possible only through a sequencing-based approach [2,17]. This involves detecting SNVs using the Sanger method, as well as identifying both SNVs and CNVs through NGS, given the use of appropriate bioinformatic tools [25,26,27]. Both Sanger and NGS-based sequencing approaches require a well-established genomic reference sequence for comparison, yet they operate at vastly different scales, from single exon to whole genome analysis. Alternatively, the multiplex ligation-dependent probe amplification (MLPA) technique, specifically the SALSA MLPA Probemix P459 for *SERPINA1*, can be used to exclude CNVs [28,29]. Therefore, identification, characterization, and clinical interpretation of novel *SERPINA1* variants is not a trivial task and requires a degree of experience.

## 4. Clinical Consequences of Pathogenic Variants in *SERPINA1*

*SERPINA1* mutations are a well-established cause of AATD. Regarding the phenotypic spectrum, AAT deficiency primarily affects the lungs, liver, and, to a lesser extent, the skin, causing diverse clinical symptoms across these organs [30]. Pathogenic variants in the *SERPINA1* gene predispose to chronic liver disorder and hepatic dysfunction (hepatitis, cirrhosis, fibrosis, cancer) from infancy to adulthood and/or to progressive obstructive lung diseases in adults, including chronic obstructive lung disease (COPD), emphysema and bronchiectasis. AATD-related liver disease affects only some of the children (manifesting as cholestatic jaundice in infants, and liver failure or cirrhosis in older children) and emphysema is very rare; nevertheless, the risk increases significantly with age. Consequently, pulmonary manifestations are predominant in adults. Affected AATD individuals are also susceptible to panniculitis and vasculitis. Development and progression of COPD is strongly affected by smoking, which accelerates and exacerbates pulmonary damage. Still, non-smokers also remain at risk of lung and/or liver disease [14,30,31].

AATD is one of the most common, yet severely underdiagnosed, rare metabolic disorders, affecting approximately 1 in 2000–5000 individuals of Caucasian descent, 1 in 5000–7000 persons from North America, and 1 in 1500–3000 from Scandinavia. Owing to the mainly adult onset of symptoms, and to sharing similar characteristics with other obstructive lung diseases (e.g., asthma) or hepatic conditions, AATD is often misdiagnosed or underdiagnosed. Also, it characterizes with variable phenotypic expression both between unrelated patients with the same alteration and within families, which makes the clinical diagnosis even more difficult. It is estimated that more than 90% of affected individuals remain not recognized, despite the high prevalence of AATD [14,30,31,32,33,34,35].

The severity of symptoms depends on the genetic background and molecular pathomechanism of the disease, associated with the resultant serum level and functional deficiency of AAT. Significant depletion of functional AAT results in dysregulation of inflammatory responses and lung tissue degradation, while accumulation of misfolded protein inclusions in hepatocytes lead to cirrhosis and liver dysfunction. Therefore, it is crucial that all patients under clinical suspicion of AATD undergo genetic testing, irrespective of their age [14,30]. Moreover, the identified genetic variants must be accurately interpreted in the context of their impact on the quantity and quality of the AAT protein, ensuring that clinical decisions are informed by a comprehensive understanding of how these variations may influence disease progression and patient outcomes.

In the lungs, AAT deficiency leads to emphysema due to an imbalance between the destructive actions of neutrophil elastase on elastin and the protective role of AAT against this degradation, a process described as a toxic “loss of function” mechanism. Factors such as cigarette smoking and infections exacerbate this imbalance by increasing the elastase activity, thereby accelerating lung tissue damage. Additionally, PI*Z antitrypsin polymers attract neutrophils, worsening inflammation and tissue destruction within the lungs [36]. Conversely, liver complications in AAT deficiency, which range from neonatal hepatitis to cirrhosis and hepatocellular carcinoma in both children and adults, arise from a toxic “gain of function” mechanism [37]. They result from the pathological accumulation of altered AAT proteins within hepatocytes, forming periodic acid–Schiff (PAS)-positive, diastase-resistant inclusions. Such liver pathology is specifically associated with genotypes leading to the abnormal polymerization of AAT within the hepatocytes’ endoplasmic reticulum, notably the PI*ZZ phenotype of AATD, caused by homozygous NM_000295.5:c.1096G>A p.(Glu366Lys) substitution [38]. The described distinction underscores the complex pathogenesis of AAT deficiency, affecting different organs through unique mechanisms.

## 5. Variant Interpretation in the Genomics Era—Future Perspective for AATD Diagnostics

Despite NGS not being widely implemented in AATD diagnostics, its advantages (high-throughput, accuracy, and low cost) might soon make it the preferred option for AATD testing, even in large-scale national screening programs [2,39]. The complexity of NGS workflows and raw data analysis is slowly diminishing with the advent of more automated pipelines, user-friendly software, and increased specialist availability; however, an expert in bioinformatics support remains crucial for the accurate handling of data output and comprehensive interpretation of the results.

The use of high-throughput sequencing, especially employing multiple-gene panels, whole-exomes, or genomes, increases the possibility of identifying a VUS and incidental or secondary findings, which may or may not have clinical relevance to the patient’s phenotype [40,41]. To accurately classify these variants, a rigorous application of the American College of Medical Genetics and Genomics and the Association for Molecular Pathology (ACMG–AMP) criteria is essential [42,43]. Also, the unified nomenclature recommended by the HGVS [8] must replace any historical variant naming. Descriptors such as “polymorphism” or “mutation” are no longer recommended for indicating clinical relevance. Instead, a comprehensive approach involving an interdisciplinary team is necessary, utilizing literature, genetic databases (such as The Genome Aggregation Database—gnomAD, ClinVar, VarSome, HGMD, Online Mendelian Inheritance in Man—OMIM, Orphanet) [7,10,13,44,45,46,47,48,49,50], computational analysis (such as in silico pathogenicity predictors), and functional studies. These efforts aim to classify variants as benign, likely benign, VUS-likely benign, VUS-likely pathogenic, likely pathogenic, or pathogenic, with only the last three categories being reportable. This underscores the importance of computational tools in verifying VUS and novel variants, as they offer a non-invasive and efficient method for predicting the potential impact of genetic alterations on protein function. In the context of *SERPINA1*, where the precise functionality and stability of the AAT protein are critical for preventing lung and liver disease, these tools are invaluable. They allow for the rapid screening of variants, facilitating early identification of potentially pathogenic changes before clinical manifestations occur. Moreover, computational analyses serve as a bridge between the discovery of genetic variants and the experimental validation of their effects, streamlining the research process and enhancing our understanding of genetic diseases. By providing a first-line assessment of variant pathogenicity, computational tools play a crucial role in the prioritization of variants for further study, ultimately accelerating the development of targeted therapies and personalized medicine strategies.

## 6. Computational Tools for Predicting the Outcome of SNVs

Among the bioinformatic tools used in modern diagnostics, a distinction should be made between tools based on gene sequence analysis and those based on protein amino acid sequence and/or protein structure analysis. The choice of appropriate tools depends largely on available data on the molecular target, including the experimentally obtained three-dimensional structure of the protein.

It is crucial to emphasize the difference between a gene’s nucleotide sequence and the amino acid sequence of the protein encoded by that gene. In transcription, the gene’s sequence information is transferred to messenger RNA (mRNA). Then, during translation, the mRNA engages with a ribosome, which translates the RNA sequence into a chain of amino acids and facilitates protein synthesis until it reaches a “stop” codon. Consequently, the gene sequence offers a more rich and complex set of information, while the amino acid sequence reveals insights into the protein’s stability, dynamics, and function [20]. In this review, we divided the computational tools into four categories: (i) tools for gene sequence analysis, (ii) tools for mutagenesis studies, (iii) tools for structure prediction, and (iv) molecular dynamics simulations. This division also reflects a step-by-step computational analysis of a gene and its variants, starting from gene sequencing, and then analyzing the native and mutated protein product, its stability, and dynamics properties.

### 6.1. Sequence-Based Computational Analysis

**Figure 1:**
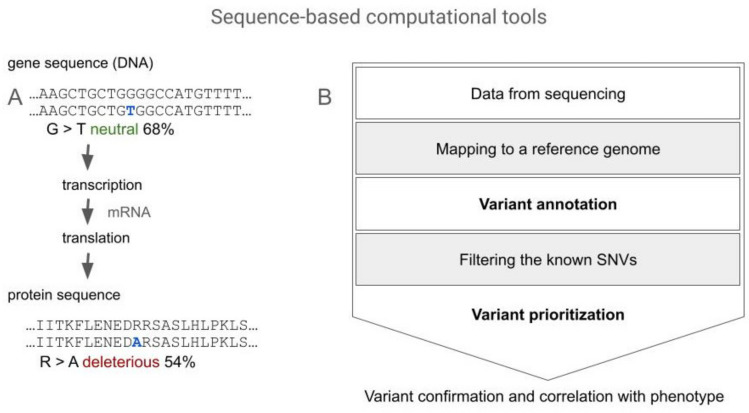
Computational tools for gene sequence analysis. The (**A**) panel shows two types of sequences which can be analyzed using computational tools and the relationship between them: the gene sequence (DNA sequence) needs to undergo transcription and translation processes to become a protein sequence (amino acids sequence). An example of the genome analysis workflow is shown in the (**B**) panel. For this review we focused on the variant annotation and variant prioritization tools, which are marked on the scheme in bold.

Sequence-based analysis is one of the fundamental approaches which involves analyzing either the gene sequence, or the amino acid sequence of the protein that is a product of gene expression. Depending on the available data, these two approaches enable prediction of the potential functional alterations, utilizing sequence conservation and changes in physicochemical properties to estimate the impact of mutations and its linkage with the disease manifestation. It is well known that a mutation in a conserved region, such as an active site cavity of an enzyme, a dimerization or binding interface, or region involved in protein–protein, protein–substrate interactions is more harmful compared to mutation occurring in a non-conserved (diverse) region [51]. For example, Shaik et al. [52] showed that missense mutations in the regions involved in forming hydrogen bonds and the RCL were highly deleterious, as per the in silico sequence analysis tools assessment. Computational tools for gene sequence analysis allow functional information to be assigned to DNA variants based on the standardized HGVS nomenclature and reducing the number of genetic variants that need to be evaluated manually. Therefore, in this review, we will divide the gene sequence-based tools into two groups: (i) tools for variant annotation and (ii) tools for variant prioritization (see Figure 1).

Variant annotation involves the systematic characterization and interpretation of genetic variants, aiming to uncover their potential functional implications and contribution to phenotypic variation. Given the landscape of tools facilitating the annotation of functional consequences attributed to genetic variants, in this review we focus only on a few of them. VEP (The Ensembl Variant Effect Predictor) [53] is a toolset provided by the Ensembl Project, which creates, integrates, and distributes reference datasets and analysis tools widely used in genomics, especially in determining the impact of genetic variation. It allows analysis and annotation of genomic variants in coding and non-coding regions. For known or overlapping variants, allele frequencies and disease or phenotype information are included, allowing users to draw conclusions on the identified changes. VEP, however, does not provide its own variant pathogenicity score, but rather integrates third party pathogenicity predictions and other relevant information from existing databases. ANNOVAR [54] aids in the identification and interpretation of potentially pathogenic molecular variants by providing comprehensive functional annotations. Key aspects of its utility in diagnostics include: (i) variant annotation, comprising information on its genomic location, potential impact on coding and/or non-coding regions, and its occurrence in known genetic databases; (ii) distinguishing between synonymous and non-synonymous changes, as well as to identify variants that may affect splicing or introduce “stop” codons; (iii) population frequency of the annotated variant, which allows clinicians to assess if it is a rare (more likely to be pathogenic) or a relatively common change (e.g., risk factor, likely benign variant) within various populations; and (iv) clinical relevance of SNVs, based on data available in genetic databases, such as ClinVar [47,48] or OMIM [45,46], providing insight into their association with known genetic diseases and aiding variant evaluation. While ANNOVAR itself is a command line Perl-based tool, wANNOVAR is a web-interface of ANNOVAR, allowing access to some of the functionalities in a user-friendly manner. FAVOR (Functional Annotation of Variants Online Resources) [55] is a whole genome variant annotation database and a variant browser that provides plenty of functional annotation scores from a variety of biological functional dimensions for all possible 9 billion SNVs and observed indels. It offers a web interface, enabling users without prior programming skills to utilize the tool in their work. Funcotator (FUNCtional annOTATOR) is an example of an integrated tool utilizing different annotation tools. It is a part of the Genome Analysis Toolkit (GATK) [56]. Funcotator enables users to annotate genetic variants based on a customizable set of criteria and various data sources, including public databases such as dbNSFP [57], ClinVar, COSMIC [58], and gnomAD [7]. The tool delivers annotated variant analysis results in a specified output file which contains detailed annotations for each variant, facilitating downstream analysis and interpretation [59].

Variant prioritization is a process that involves selecting and ranking variants based on criteria such as predicted functional impact, allele frequencies, and relevance to the phenotype or disease under study. There is a plethora of tools that facilitate achieving the mentioned goal, hence only a select few are described in this review. VPOT (Variant Prioritization Ordering Tool) [60] is a Python-based command line tool that allows researchers to create a fully customizable pathogenicity ranking score from any number of annotation values, each with a user-defined weighting. VPOT also provides functionalities to allow variant filtering based on a candidate gene list or by affected status in a family pedigree. REVEL (Rare Exome Variant Ensemble Learner) [61] is a metapredictor that integrates predictions from various individual tools and databases, utilizing ensemble learning techniques to enhance overall performance. REVEL considers multiple features, such as conservation scores, functional annotations, and population allele frequencies, to provide a comprehensive prediction of variant pathogenicity. Giacopuzzi et al. [62] showed that REVEL performed best in discriminating variants of known pathogenicity from those classified as benign for coding *SERPINA1* variants. This tool was assessed as having the highest potential for clinical utility in in silico evaluation of new *SERPINA1* variants. PERCH (Polymorphism Evaluation, Ranking, and Classification for a Heritable Trait) [63] and VarSome [49] are integrated frameworks allowing users to prioritize genetic variants. The main advantages of such platforms are their ability to streamline workflows, offering efficient access to varied information. These frameworks contribute to improved data accuracy, time saving, and a more comprehensive understanding of genetic variants. PERCH is a framework tailored for the analysis of NGS-derived genetic variants. This platform integrates a novel pathogenicity metric, BayesDel, which merges Bayesian methods with additional allele frequency data. It enhances assessment of the biological relevance of genes to diseases, adjusts linkage analysis, introduces a rare-variant association test, and refines the variant call quality score. It is helpful in estimating the potential impact of novel SNVs of unknown significance by employing a quantitative integration of allele frequencies, deleteriousness, association, and co-segregation. VarSome, a comprehensive platform for variant interpretation, employs multiple metapredictors to consolidate and refine their predictions, providing a more extensive assessment of the impact of genetic variants, enhancing the accuracy and reliability of the predictions [49]. Furthermore, it is a search engine for human genomic variation, both germline and somatic, aggregating data from multiple genetic databases, among others, on variant nomenclature (according to HGVS), genomic localization, frequencies in various populational databases (like gnomAD), known associations with genetic disorders (e.g., according to OMIM), and potential clinical significance (e.g., ClinVar). Importantly, VarSome implements the ACMG–AMP criteria to assess SNVs, simplifying their usage in the clinical setting. Users have the ability to reclassify the pathogenicity assessment of the queried variant, according to the additional clinical and genetic data available to them, and their expertise. VarSome builds a community specialized in the field of medical genetics, sharing their global knowledge to the benefit of understanding the impact of human variation on genetic diseases. Some tools may also be used for both variant annotation and prioritization, such as the Genome-Environment-Trait Evidence (GET-Evidence) system, which automatically processes genomes and prioritizes both published and novel variants for interpretation [64]. In terms of rare diseases, Licata and colleagues have written an exhaustive guide on how to improve variant prioritization in rare disorders. [65]. Comprehensive reviews covering differences between concept, usage, and accuracy of various variant prioritization tools were written by Katsonis et al. [66] and Ghosh et al. [67]. Over the years, tools for genome interpretation have been assessed by CAGI—Critical Assessment of Genome Interpretation. Conclusions summarizing the first 50 challenges, spanning over 10 years of the CAGI experiment, have been recently published [68]. In those challenges, the participants made blind predictions of phenotypes based solely on the genetic data, and their predictions were then assessed by independent judges. It was shown that while current methods for genome interpretation are not perfect, they have major utility in scientific and clinical usage, and that combining methods and creating large, robust datasets for training and assessment of the tools promise future progress of the mentioned tools.

Tools for assessing variant pathogenicity based solely on the gene sequence vary from simple annotating tools to complex integrated frameworks. Analysis of a gene sequence and identification of novel variants is a first step on the long path of understanding a particular disease. While helpful and powerful, the sequence-based computational tools need more reliability and accuracy. However, a good practice is combining the results of several similar tools and cross-correlating them. For example, in the previously mentioned work by Shaik et al. [52] they used VEP to access predicted pathogenicity scores provided by six different variant pathogenicity predictors, namely Combined Annotation Dependent Depletion (CADD), Scale-invariant Feature Transform (SIFT) [69], Polymorphism Phenotyping (PolyPhen) [70], Mendelian Clinically Applicable Pathogenicity (M-CAP) [71], Functional Analysis through Hidden Markov Models (FATHMM) [72], and REVEL [61]. Interestingly, all tools assessed only one *SERPINA1* variant (NM_000295.5:c.833T>C p.(Leu278Pro), rs1566753480:A>G) as pathogenic. In another work by Tuteja et al. [4], the authors benchmarked three tools, namely Alamut^®^ Batch, VEP, and ANNOVAR, against 298 variants from the medical exome database at the Molecular Diagnostics Laboratory at Lurie Children’s Hospital, including *SERPINA1* variants. The test set of 298 intronic and exonic variants across 191 genes, previously classified and reviewed in clinical reports by the Molecular Diagnostics Laboratory at Lurie Children’s Hospital, was curated and used for their study. These variants were generated by targeted sequencing of ~4700 genes (so-called “medical exome”) from 105 patients. Based on their results, VEP was the most accurate (correctly predicted 297 out of 298 variants), Alamut^®^ Batch was second best (296 out of 298), while ANNOVAR was only able to annotate 278 out of the 298 variants correctly. Another interesting study was conducted by Rigobello et al. [73] on four families from the AATD Italian Registry with siblings concordant for the genotype, but with discordant phenotypes (emphysema/no emphysema). For variant filtering and prioritization they used QueryQR [74], showing that gene variants contributing to the development or suppression of AATD were mainly involved in the regulation of immune homeostasis and the maintenance of self-tolerance.

As a product of gene expression, the protein sequence is also a subject of valuable analyses, for which the following tools can be used: MutPred [75], nsSNPAnalyzer [76], PolyPhen-2 (PPH-2) [70,77], SNAP [78], MAPP [79], PANTHER [80], PhD-SNP [81], SIFT [69], SNPs&GO [82], and the previously mentioned VEP webserver. They are primarily designed to evaluate whether the provided mutation is neutral or pathogenic (Figure 1A). Similarly to the gene sequence-based tools, it has been shown that using a combination of some of these tools to provide a consensus prediction comes with significantly increased accuracy of prediction [83]. An example of such a consensus prediction tool is PredictSNP [84] which integrates MAPP, nsSNPAnalyzer, PANTHER, PhD-SNP, PolyPhen-1 [85], PolyPhen-2, SIFT, and SNAP tools, and assesses the impact of mutations by assigning a pathogenicity prediction together with a score of confidence to the provided variant. In the work by Denden et al. [86], the authors used SIFT software, and PolyPhen and HybridMeth algorithms to assess the effect of a novel NM_000295.5:c.169T>C p.(Phe57Leu) (rs1457464431:A>G) mutation on AAT protein. The phenylalanine residue was shown to be conserved, and thus it was also shown to be less stable compared to the native protein. Moreover, PolyPhen results suggested that substitution of the buried phenylalanine by leucine at position 57 reduced the cavity size in the protein structure and thus it is very likely to disturb the tridimensional structure and folding of AAT. Nevertheless, it must be kept in mind that protein structure predictions may be inaccurate, especially in the case of proteins that are switching their conformation and/or have an unstructured, disordered loop within their structure as AAT. The tools for assessing the outcome of a molecular variant based on protein sequence suffer from the same problems as tools based on gene sequence. Using several of them is good practice, and further validation, especially experimental, is always beneficial.

### 6.2. In Silico Protein Stability Analysis

As the initial prediction of the outcome of a gene variant has been described in the previous section, the next step is to introduce a mutation into the protein structure. In a perfect scenario, the three-dimensional structure of the gene product is already known and deposited in the Protein Data Bank (PDB) database [87]. In the case of the *SERPINA1* gene product, several low resolution structures of the AAT protein are actually deposited in the database, including the intact native protein (PDB ID 3ne4) [88], a native protein in complex with a protease (PDB ID 1ezx) [89], AAT in dimeric (PDB ID 2znh) [90] and trimeric form (PDB ID 3t1p) [91], or some of the well-known deleterious variants, such as the PI*Z variant (PDB ID 5i01) [92]. However, for the less known proteins their crystal structure may not be deposited yet; therefore, this step should be followed by a protein structure prediction, as described in the following section.

**Figure 2:**
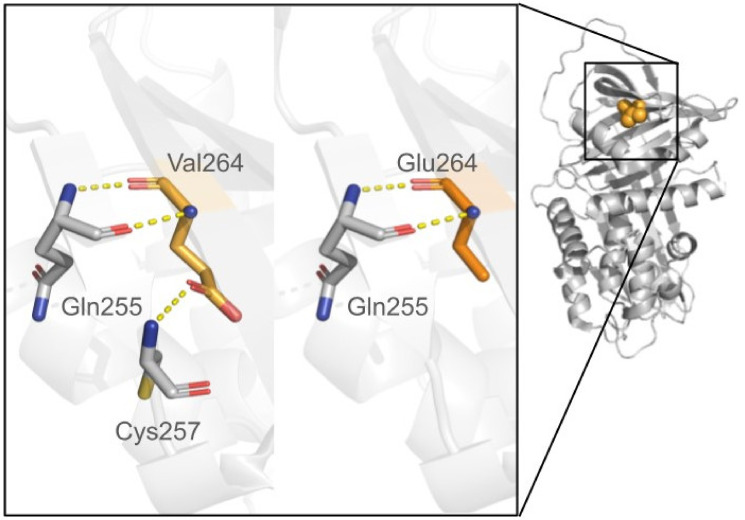
Schematic representation of the mutagenesis study of alpha-1 antitrypsin with introduced p.Glu264Val substitution (HGVS: NM_000295.5:c.863A>T p.(Glu288Val) or rs17580:T>A; described as PI*S variant). The introduced mutations are shown as orange sticks with the hydrogen bond network shown as yellow dashed lines, the adjacent residues are shown as grey sticks; nitrogen atoms are blue, oxygen atoms—red, and sulfur atom—yellow. The mutagenesis studies were carried out by the authors.

In silico protein stability analysis can be performed using computational tools which introduce mutations into an already known protein structure (as shown in Figure 2) or a protein model predicting the impact of that mutation on stability and binding affinity by computing thermodynamic parameters, such as the alteration in folding free energy (ΔΔG). Mutation can be assessed as pathogenic if it destabilizes the protein, i.e., the ΔΔG values of the variant protein are higher compared to the native protein. On the other hand, an altered protein can exhibit ΔΔG values lower than the native protein, thus being more stable. A good overview of the existing tools for protein stability assessment has been provided elsewhere [93,94,95]. A different approach to assess pathogenicity of a molecular variant was proposed by the authors of the AlphaMissense tool [96]. AlphaMissense is a deep learning model that is an adaptation of AlphaFold (AF) for predicting missense variant pathogenicity. While it takes an amino acid sequence as an input, it predicts the pathogenicity of all possible single-amino acid changes at a given position in the sequence as a scalar value. AlphaMissense does not predict the structural changes of the mutated amino acid sequences but incorporates structural context by using an AF-derived system with evolutionary context. The model was trained on the population frequency data, and evaluated against related methods using clinical databases not included in the training dataset [96]. The results showed agreement with combined assays of variant effect assessment. Furthermore, predictions were performed for all single–amino acid substitutions in the human proteome and are community available.

It is worth noting that both variants with higher or lower stability may display a pathogenic outcome. Some proteins require a particular degree of freedom and flexibility to fulfill their physiological role; therefore, increased stability of the protein variant may impair its function. For example, Kueppers et al. [16] used FoldX [97] combined with the previously described PolyPhen and support vector machine (SVM) program [98] to assess the utility of NGS and predictive computational analysis to guide the diagnosis of patients suspected of AATD. They also mapped mutations to the AAT protein structure and thus suggested the pathogenicity mechanism for the most pathogenic variants causing disruption of the packed hydrophobic core of AAT. Furthermore, they compared their results with the previous analysis by Giacopuzzi et al. [62] who also evaluated the utility of several computational tools for *SERPINA1* variants’ analysis. Both authors raise an important point, that several computational tools should be consulted in the clinical decision-making. It is also beneficial to cross-validate the results of a mutagenesis study with the previously described sequence-based tools. Of note, in a vast majority of *SERPINA1*-related research, for simplification’s sake, the information on the base alleles and interactions between the native and mutated protein are neglected, in order to focus solely on the biophysical impact of the mutation on the protein stability and function.

### 6.3. Homology Modeling and Structure Predictions

**Figure 3:**
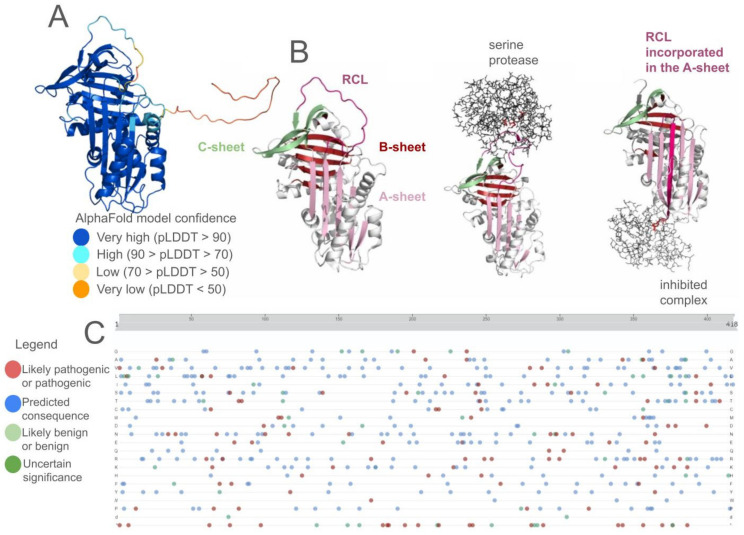
The complexity of the alpha-1 antitrypsin (AAT) structure. (**A**) AlphaFold model of the AAT protein (based on the UniProt ID P01009). The AlphaFold model is created based on the full sequence of the protein, thus it consists of an additional N-terminal part. (**B**) Mechanism of inhibition: after proteolytic cleavage of the reactive center loop (RCL), the loop incorporates into the A-sheet in the protein structure and the protease is inhibited. (**C**) A screenshot from the UniProt database (ID P01009) of the known variants of AAT. The viewer provides 502 variants from UniProt as well as other sources including ClinVar and dbSNP.

Structure prediction is a valuable technique that can help with obtaining three-dimensional protein structures in the case of a lack of experimentally derived structures or missing fragments of the protein of interest (Figure 3). The PDB database provides access to both experimentally obtained protein structures and Computed Structure Models (CSM) from AlphaFold DB [99] and RoseTTaFold ModelArchive databases [100]. In this review, we will divide structure prediction approach into two groups: (i) homology modeling and (ii) ab initio structure prediction. In homology modeling, a sequence alignment is completed prior to structural template matching of homologous proteins. This allows researchers to construct proper atomic-level models of the protein for further analysis. Such an approach has been widely used in rare disease research [101], when the protein of interest is not well known or its three-dimensional structure is not deposited in the PDB database. SWISS-MODEL [102] and MODELLER [103] are some of the tools utilizing this approach. In addition, recent breakthroughs in protein structure prediction have been exemplified by AlphaFold, an artificial intelligence (AI)-driven tool developed by DeepMind [99] or RoseTTaFold [104]. AlphaFold has garnered immense attention in the scientific community for its remarkable ability to predict protein structures based on the protein sequence with unprecedented accuracy. Recently, attempts have been made to harness AlphaFold’s structure prediction capabilities to determine the effect of SNVs [96,105]. Efforts to apply this approach to investigate mutations in proteins belonging to the SERPIN family did not give the expected outcomes, as AlphaFold tended to generate native structures for all variants, irrespective of their complexity and potential in vivo conformational effects [106]. Hence, there is a clear imperative for research to enhance the accuracy of AlphaFold’s predictions regarding the structural implications of SNVs in AAT and other proteins that maintain their function via conformational changes.

### 6.4. Molecular Dynamics Simulations

**Figure 4:**
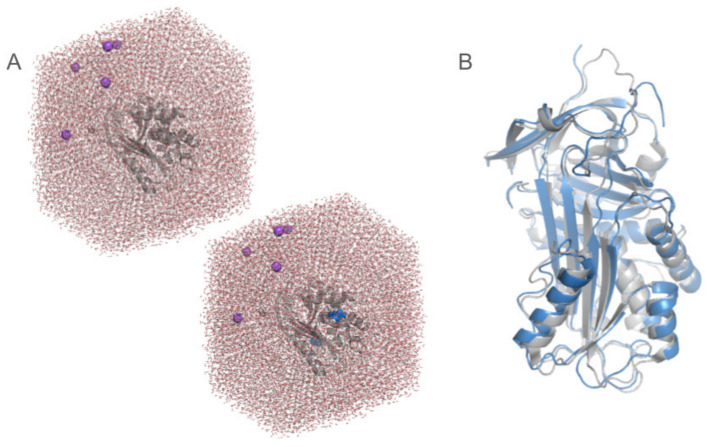
Schematic representation of molecular dynamics (MD) simulations setup. (**A**) Starting point system for the MD simulations of the native protein (**top**) and the variant protein (**bottom**, mutation shown as blue spheres). MD simulations are conducted in the presence of water molecules and ions. Protein structure is shown as grey cartoons, ions as purple spheres, and water molecules as red (oxygen) and white (hydrogen) lines. (**B**) MD simulations of alpha-1 antitrypsin (AAT) in which the reactive center loop (RCL) is cleaved in order to simulate the folding mechanism. The crystal structure of the AAT protein is shown as white cartoons and the samples snapshot of the simulation as blue cartoons. For clarity, water molecules and ions were removed. Snapshots presented on both panels come from actual simulations conducted by the authors.

Molecular dynamics (MD) simulations offer a dynamic, atomic-level perspective on protein behavior, allowing researchers to gain insight into the complexities of mutant protein variability [21,107]. Through the use of Newton’s equations of motion, MD simulations imitate the movement of atoms within a protein over time. Selected tools for performing MD simulations are Gromacs [108], AMBER [109], NAMD [110], and Charmm [111]. One of the primary advantages of MD simulations is their ability to capture changes in the protein conformation caused by mutations. By running simulations for both native and mutant protein structures, researchers can observe how mutations alter the protein’s three-dimensional structure dynamics [112] and affect protein–protein interactions [113], cause protein aggregation [114] or impair enzymatic activity [115], all of which can be linked to disease mechanisms. In AAT research, MD simulations were used to study conformational changes during the protein’s inhibitory function [116], or investigate the pathogenic PI*Z variant causing protein aggregation [117]. MD simulations provide significant amounts of various data; therefore, it is crucial to know the properties one may want to investigate before running the simulations (Figure 4). Moreover, MD simulations are computationally demanding and require significant computational resources. The accuracy of simulations depends on multiple factors, like the quality of the protein structure model, force field parameters, and simulation time scales, thus requiring experience to obtain a proper model of desired phenomena. Additionally, it is worth noting that, for clarity, some of the structural features may be neglected, for example in most of the MD simulations (or mutagenesis studies), the glycosylation regions are neglected. Khan et al. [118] presented a detailed protocol on parameterizing the glycosylation regions and then running MD simulations. They confirmed that glycated proteins are more structurally stable compared to aberrant glycated proteins in both MD simulations and in experiments. They also noticed that most of the naturally occurring proteins are glycosylated, but only 4% of X-ray crystallographic structures in the Protein Data Bank (as of March 2021) include covalently bonded N- or O-glycans. They suggest that the reason is that most target proteins are partly or completely truncated before crystallization to eliminate the glycosylation. Also, similarly as in the case of mutagenesis study, the information on the base alleles is generally neglected for MD simulations. Moreover, the aggregation process is difficult to study, as in MD simulations only a single protein is simulated at a time. Importantly, validation through experimental studies (e.g., using protein activity assays, on model organisms) and complementary bioinformatic techniques is necessary to increase the reliability of in silico computations [10,16,19,22,42,43,62,119].

## 7. Concluding Remarks

A comprehensive understanding of the impact of SNVs in the *SERPINA1* gene on protein structure and function requires integration of diverse bioinformatic methods. Here, we reviewed computational tools that may be used to describe a novel molecular variant. Even though we focused on *SERPINA1* and AAT, the review is universal and provides a step-by-step guide for clinicians on how to report novel variants using computational approaches.

The initial phase in creating a comprehensive report on a new variant involves utilizing sequence-based tools for analyzing both the gene and protein sequences. These tools range from basic command line tools to more complex integrated systems. The variant annotation and variant prioritization tools, as well as their application for *SERPINA1* studies, were shown in regard to the gene sequence analysis. It is recommended practice to validate these tools by applying multiple methods to seek a unified result. Given the high number of VUS reported for *SERPINA1*, incorporating experimental validation can provide additional benefits.

The second step of this guide depends on the availability of a three-dimensional structure of a protein of interest. Ideally, a good quality crystal structure is available and can be used as a template for further analysis, such as mutagenesis studies and MD simulations, as for AAT protein. However, in the scenario when the structure of the protein remains unknown, additional approaches for protein structure prediction are available. The most recent cutting-edge approaches involving machine learning techniques have proven their accuracy in predictions, but it is important to keep in mind their limitations. For example, AlphaFold does not predict the positions of bound ligands, such as heme in hemoglobin, and therefore the AlphaFill was created [120]. Another limitation is that at the beginning it was able to predict only a single structure, and thus some solutions were provided to obtain multiple conformations of the predicted models [121]. It is worth noting that protein structure prediction methods are unable to predict an outcome of indel mutations or CNVs accurately. Frameshift mutations are also a challenging problem, due to the fact that they may affect a bigger fragment of a protein sequence than a simple SNV, generally leading to protein truncation. In the case of a known protein structure deposited in the PDB database or an accurate protein model, mutagenesis study can be employed; however, it has the same limitations as the structure prediction approach. While it is limited only to SNVs, it also neglects the effect of glycosylation and other post-translational modifications. It is also worth noting that in the mutagenesis studies, the pathogenicity is in most cases assessed based on the differences in ΔΔG values between the native protein and the altered protein. While higher ΔΔG values indicate destabilization of the variant protein, and thus pathogenic effect, lower ΔΔG values do not necessarily indicate a benign outcome. Proteins require a particular degree of freedom, and thus increasing its stability may also result in a pathogenic effect. Also, it is good practice to cross-correlate the mutagenesis results with some other tools, including the sequence-based tools.

Finally, to describe a novel variant in detail, it is recommended to run several MD simulations of the variant protein against the native protein. However, this approach requires a good structural model and knowledge of the protein itself. The detail level and accuracy of the simulations can be increased by applying a set of system-specific modifications, such as adding glycans to the glycosylation sites or including base alleles. Importantly, MD simulations are usually applied to a single protein, and thus the aggregation process may be challenging to simulate, despite being a major pathomechanism of some disorders, such as AATD. This also implies that the effect of compound heterozygotes, namely different mutations occurring on different alleles, cannot be easily established. Again, compound heterozygotes are often a case in diseases which are inherited in an autosomal codominant pattern, such as AATD. Eventually, specific techniques for analysis of MD simulations, such as PCA-based conformational changes analysis [118], can also be applied to enhance the obtained results and observations.

Insights from computational studies are crucial not only for deciphering the molecular basis of disorders related to AATD, but also for targeted therapies and customized treatment plans that align with individual genetic makeup. It was also shown that the machine learning techniques can be applied for rare diseases analysis [122]. Combining genomic, proteomic, and computational data is a promising approach for advancing precision medicine, with a specific focus on the potential of gene therapy and small molecule interventions to correct or restore AAT functionality.

## Data Availability

Not applicable.
